# Hydroxyl Ion Diffusion through Radicular Dentine When Calcium Hydroxide Is Used under Different Conditions

**DOI:** 10.3390/ma11010152

**Published:** 2018-01-17

**Authors:** Michael Cai, Paul Abbott, Jacqueline Castro Salgado

**Affiliations:** UWA Dental School, The University of Western Australia, Crawley, WA 6009, Australia; michaelhcai@gmail.com (M.C.); jacky.castro-salgado@uwa.edu.au (J.C.S.)

**Keywords:** calcium hydroxide, diffusion, Ledermix paste, endodontics, root canal treatment

## Abstract

Calcium hydroxide’s anti-bacterial action relies on high pH. The aim here was to investigate hydroxyl ion diffusion through dentine under different conditions. Teeth were divided into control (*n* = 4) and four experimental groups (*n* = 10): Group 1—no medicament; Group 2—Calmix; Group 3—Calmix/Ledermix; Group 4—Calasept Plus/Ledermix; Group 5—Pulpdent/smear layer. Deep (inner dentine) and shallow (outer dentine) cavities were cut into each root. pH was measured in these cavities for 12 weeks. The inner and outer dentine pH in Group 2 was significantly higher than all groups. Inner dentine pH in Group 3 was slightly higher than that in Group 4 initially but subsequently comparable. After Day 2, Group 5 had significantly lower pH than Groups 3 and 4. The outer dentine pH in Group 3 started higher than that in Groups 4 and 5, but by Day 28 the difference was insignificant. The time for the inner dentine to reach maximum pH was one week for Group 2 and four weeks for Groups 3 and 4. The time for the outer dentine to reach maximum pH was eight weeks for all experimental groups. Mixing different Ca(OH)_2_ formulations with Ledermix gave similar hydroxyl ion release but pH and total diffusion was lower than Ca(OH)_2_ alone. The smear layer inhibited diffusion.

## 1. Introduction

The main aim of root canal treatment of infected teeth is to disinfect the root canal system. However, this cannot be reliably achieved by root canal instrumentation with files and irrigation with anti-bacterial solutions, so the supporting action of intracanal medicaments is required [1,2,3]. Byström and Sundqvist reported that they were able to cultivate bacteria from the root canals of 28% of the tested teeth, even after three visits of root canal instrumentation and irrigation with sodium hypochlorite (NaOCl), ethylenediaminetetraacetic acid (EDTA), or a combination of these irrigants [1]. They also reported an increase in bacterial numbers between appointments when no anti-bacterial medicament was placed. Therefore, the use of inter-appointment antibacterial medicaments in the root canal system is necessary to predictably achieve bacteria-free root canals during endodontic treatment [1,2,3].

Calcium hydroxide (Ca(OH)_2_) is one of the most popular root canal medicaments and it comes in different forms and combinations [4]. Ca(OH)_2_ has several roles during root canal treatment, namely, (A) antibacterial action [5], (B) the ability to stimulate hard tissue formation (e.g., apexification [6] and apexogenesis [7]), (C) the ability to dissolve tissue [8], and (D) the ability to cause intratubular mineralization in dentine [9]. The anti-bacterial action is a direct effect of the hydroxyl ion on micro-organisms, the interaction of Ca(OH)_2_ with carbon dioxide, which alters the local environment for bacteria [10], and the ability of the hydroxyl ion to break down necrotic tissue [8,11,12], which may act as a substrate for bacteria. Ca(OH)_2_ has a high pH as a result of the release of hydroxyl ions. 

Previous studies have demonstrated that Ca(OH)_2_ pastes when placed in the root canal system release the hydroxyl ion, which then diffuses through the root dentine and cementum to reach the peri-radicular tissues [13,14]. An important aspect of this diffusion process is to deliver the hydroxyl ion throughout the entire root canal system—that is, to all the dentine tubules, fins, transverse anastomoses, isthmuses, lateral canals, and accessory canals—since bacteria may be present in any or all of these sites. Increasing the pH in the dentine can therefore inhibit and destroy bacteria that remain following the mechanical procedures (instrumentation) and irrigation of the root canal during treatment. A previous study [13] showed that pH levels in the dentine vary according to location with inner dentine levels (i.e., adjacent to the root canal), reaching a peak of 10.8 within a few hours and then stabilizing at about pH 10.0. In the outer dentine, pH changes were much slower, taking up to three weeks to stabilize at pH 9.3. In that study, samples were only tested over a four-week period [13]. Hence, it is not known whether these pH levels are maintained in the dentine when long-term dressings are used during root canal treatment.

Commercial preparations of Ca(OH)_2_ are available as pastes using either saline (e.g., Calasept Plus—Nordiska, Ängelholm, Sweden), methyl cellulose (e.g., Pulpdent paste—Pulpdent Corporation, Watertown, MA, USA), or polyethylene glycol (PEG) (e.g., Calmix—Ozdent Pty Ltd., Castle Hill, Australia) as the base vehicle for the paste.

Ledermix paste (Haupt Pharma GmbH, Wolfratshausen, Germany) is a popular medicament in many countries [2]. It contains demeclocycline and triamcinolone as its active components. The former is a tetracycline antibiotic, which is designed to inhibit bacterial growth within the root canal system; the latter is a corticosteroid used to reduce inflammation in any remaining pulp tissue and within the periapical tissues [2]. The use of calcium hydroxide and Ledermix paste together as an inter-appointment medicament has been shown to have great promise in achieving a bacteria-free environment as well as reducing pain [3,15,16,17] and for the control of external inflammatory root resorption in trauma cases [18]. The effects on hydroxyl ion release and diffusion when Ledermix paste is mixed with Ca(OH)_2_ pastes has not been investigated previously.

A smear layer is created when the root canal walls are instrumented with files [19,20]. Whilst this smear layer can be removed by using chelating agents as irrigants (such as EDTA) [19], not all clinicians remove it, even though studies have shown that its presence significantly reduces the ability of the tetracycline and corticosteroid components of Ledermix paste to diffuse through root dentine [20]. Hydroxyl ion diffusion was also inhibited in one study, although it was only tested over a seven-day period [19]. If the medicaments cannot diffuse effectively through dentine, then the anti-bacterial action of the material is likely to be reduced.

The main aim of this study was to compare hydroxyl ion release and diffusion through root dentine by measuring the pH when different formulations of Ca(OH)_2_ are used in combination with Ledermix paste. A second aim was to investigate whether smear layer on the root canal walls following root canal preparation affected diffusion of hydroxyl ion through dentine.

## 2. Results

### 2.1. Inner Dentine pH

**Figure 1:**
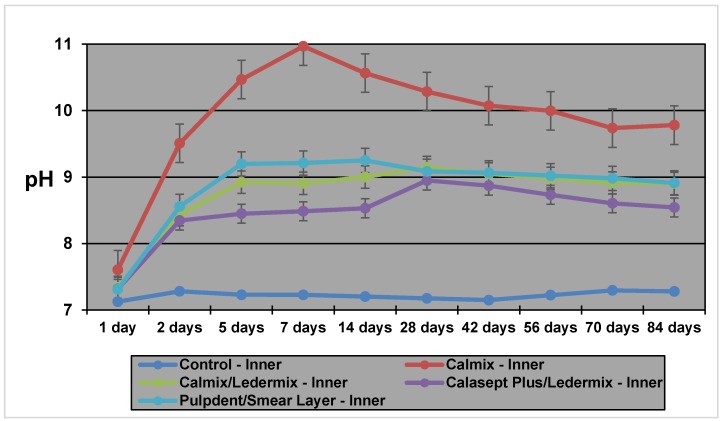
The pH values in the inner dentine with the various medicaments over 12 weeks.

Table 1 and Figure 1 show that the inner dentine pH of Calmix was significantly higher than all the other medicament groups for most of the experimental period of 12 weeks (84 days). This group started by having marginally higher pH than the other groups and it reached a peak on Day 7. The differences decreased from this point on, but they were still much higher at the final time point than the other groups. Of the other three groups, the pH of the Calmix/Ledermix group was slightly higher than the Calasept Plus/Ledermix group for Days 5–14, but subsequently they were comparable. The Pulpdent/smear layer group had significantly lower pH levels than both the Calmix/Ledermix and Calasept Plus/Ledermix groups.

### 2.2. Outer Dentine pH

**Figure 2:**
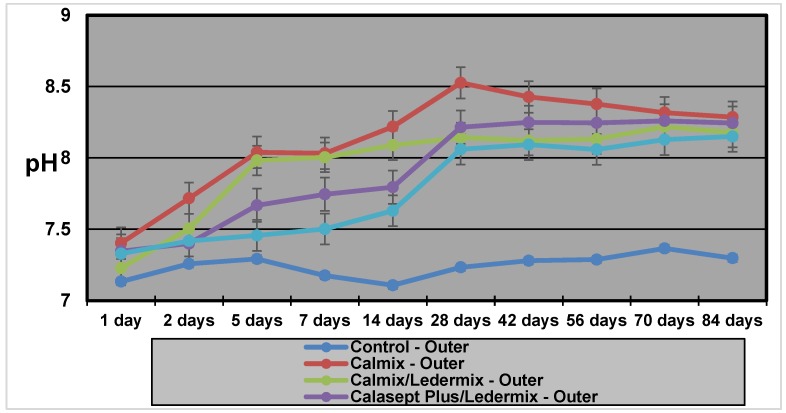
The pH values in the outer dentine with the various medicaments over 12 weeks.

Table 1 and Figure 2 show that the outer dentine pH for the Calmix group was significantly higher than all the other groups for most of the experiment. However, after 8 weeks, the difference in pH was much smaller when compared to the Calmix/Ledermix and Calasept Plus/Ledermix groups. The pH of the Calmix/Ledermix group started at a higher level than the Calasept Plus/Ledermix group and the Pulpdent/smear layer group. However, by Day 28, the difference was no longer statistically significant.

### 2.3. T_max_ Comparisons

The Calmix group exhibited a higher pH than the both the Calmix/Ledermix and Calasept Plus/Ledermix groups at all time intervals. The T_max_ (time taken to reach maximum pH) for Calmix in the inner dentine was one week compared to four weeks for both the Calmix/Ledermix and Calasept Plus/Ledermix groups. The T_max_ in the outer dentine was eight weeks for all groups apart from the control group (10 weeks), but the controls had no real increase in pH from the baseline levels.

### 2.4. Area under the Curve Analysis

**Figure 3:**
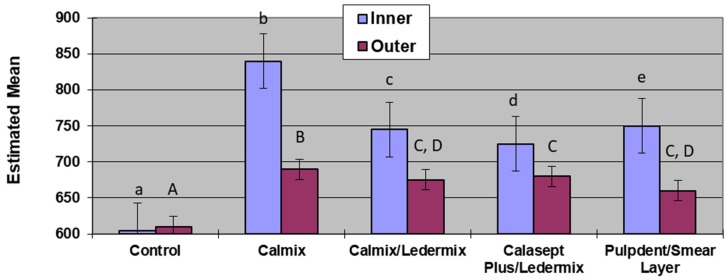
Area under the curve comparisons between groups. Groups with the same letter were not statistically significantly different from each other (lower case for inner dentine; upper case for outer dentine).

Analysis of the area under the curve across the different medicament groups (Table 2 and Figure 3) allowed the total release of hydroxyl ions to be compared. The control group, as expected, was significantly lower than all other groups in both the inner and outer dentine. Calmix had the highest amount of total release of hydroxyl ions for both the inner dentine and outer dentine measurements, and these were statistically significantly higher than the other experimental groups. The medicament group combinations that were not statistically significantly different from each other were the outer dentine measurements of the Calasept Plus/Ledermix group and the Calmix/Ledermix group, as well as the Calmix/Ledermix group compared with the Pulpdent/smear layer group.

## 3. Discussion

The pH measurements in this study demonstrate that hydroxyl ions are readily released from the various Ca(OH)_2_ formulations, even when mixed with Ledermix paste. They then diffuse through the root dentine. The results and diffusion patterns were consistent with other similar studies that have investigated pH changes in root dentine [13,14].

Calmix, a Ca(OH)_2_ paste with a 30% PEG base had higher maximum pH values in both the inner (11.27) and outer dentine (8.92) than the methyl cellulose-based Pulpdent paste (10.0 and 8.6 respectively for inner and outer dentine) that was investigated in a similar model in a previous study [14]. The time taken to reach the maximum pH (T_max_) in the inner dentine was faster for Calmix (1 week) than for Pulpdent (23 days) in the previous study. However, Calmix took 8 weeks to reach maximum pH in the outer dentine compared to 36 days for Pulpdent in the previous study [14]. The different paste base may explain the slower release over time for the Calmix group.

There was no difference in the maximum pH values or the T_max_ for the two groups where Ledermix paste was mixed with two different forms of Ca(OH)_2_. However, with the variability obtained in the different groups for the T_max_, it is advisable to increase the length of time between treatment appointments when Ca(OH)_2_ and Ledermix pastes are used together in order to ensure that the maximum pH has been obtained in the outer dentine when treating infected root canal systems.

Results for the Pulpdent/smear layer group were consistent with the results found by Foster et al. [19] although the methodology was different. The statistical analyses of both studies show that the presence of the smear layer on the root canal wall covering the dentinal tubules limited the diffusion of hydroxyl ions into the dentine tubules, and this resulted in both slower diffusion rates and lower pH in both the inner dentine and outer dentine. Therefore, the use of EDTAC during root canal preparation to remove the smear layer facilitates the maximum therapeutic effects of calcium hydroxide in the dentine and root canal system [1,19]. This is a similar finding to the effects of the smear layer on the diffusion of the active components of Ledermix paste through root dentine [20].

The model used in this experiment was an adaptation of that reported by Nerwich et al. [13] with the main modification being the measurement of pH at the mid-root level only rather than in the coronal and apical thirds of the roots. This modification was adopted following another similar experiment by Plataniotis and Abbott [14], who showed similar patterns of pH changes in mid-root cavities as that reported by Nerwich et al. [13]. Hence, the simpler experimental design was adopted.

Nerwich et al. [13] reported that the pH changes took up to 7 days to reach their peak in the outer dentine and then another 2–3 weeks to reach their maximum values of 9.3 and 9.0 in the cervical and apical thirds, respectively. The pH in the inner dentine showed more rapid changes, being evident within a few hours and peaking at 10.8 in the cervical third and 9.7 in the apical third. In that experiment, measurements were only done for 28 days and their data indicated slight rises in pH were still occurring at the end of the experiment. Nerwich et al. only tested one formulation of Ca(OH)_2_ paste, which had a saline base. Plataniotis and Abbott [14] tested different forms of Ca(OH)_2_—that is, a saline-based paste, a methyl cellulose-based paste, Ca(OH)_2_ powder mixed with saline, and a gutta percha point impregnated with Ca(OH)_2_. They reported similar diffusion patterns and pH values for the three aqueous solutions. However, the gutta percha-impregnated points did not release any significant amount of hydroxyl ion as pH values were not statistically different to the saline controls. Only the mid-root dentine was tested, and the measurements were taken over a 12-week period. The current study was also limited to this time frame, which is consistent with clinical recommendations to renew intracanal dressings every three months if long-term treatment is being provided [3].

The higher pH values obtained when Ca(OH)_2_ was used alone may be beneficial to its bactericidal activities, although it also poses a higher potential for damaging the periodontal ligament cells [2]. Taylor et al. [17] showed that a 50:50 mixture of Ca(OH)_2_ and Ledermix pastes retained the properties that were thought to be of therapeutic benefit whilst not increasing the toxicity of the component parts to mammalian cells [17]. Although slightly lower pH levels were achieved in the dentine by mixing Ca(OH)_2_ with Ledermix paste, the difference was small, particularly in the outer dentine, so it is likely to still have bactericidal effects.

## 4. Materials and Methods

Forty-four single-rooted extracted human maxillary and mandibular canine teeth with a single root canal were used. The teeth were collected from the Dental School’s collection of teeth that are used for teaching and research purposes. Such teeth are collected under the approval of the University’s Human Research Ethics Committee. The root canal was assessed radiographically to determine suitability. Teeth were initially stored at room temperature in a solution of 0.9% buffered saline. 

After standard endodontic access cavities in all teeth were cut, the root canals were instrumented and prepared using Hedström files to various sizes, according to the initial canal size and anatomy of the particular tooth. Irrigation was performed with a combination of 1% NaOCl (EndoSure Hypochlor—Dentalife, Ringwood, Australia) and 17% EDTAC (EDTA with 0.85% Cetrimide) (Colgate/Orapharm, Victoria, Australia) during instrumentation. After the use of a file in the canal, the canal was irrigated with 2 mL of NaOCl or EDTAC on an alternating basis, except in Group 5 where the smear layer was intentionally created and left in place—this was achieved by only irrigating with NaOCl and not using EDTAC at all.

All experimental and control teeth had two cavities cut into the external root surface at the mid-root level with a size 6 flat fissure bur in a low-speed handpiece. One cavity was shallow—that is, just into the dentine on the buccal root surface (outer dentine). The other cavity was cut deep into the dentine on the lingual surface of each root (inner dentine). The outer dentine cavity was cut to an approximate depth of 0.5 ± 0.25 mm from the buccal root surface. The inner cavity was cut to as deeply as possible so that it would be close to the canal walls without penetrating into the canal. The pre-operative radiographs were used as a guide to determine the depth of these cavities. The smear layer in these cavities was removed with a four minute application of 17% EDTAC. Once the teeth had been prepared in this manner, they were stored in unbuffered saline, and this solution was changed on a weekly basis throughout the experimental period. 

The teeth were divided into four experimental groups (*n* = 10) and a control group (*n* = 4). The distribution of teeth into the groups was done such that an equal number of each tooth type was present in each experimental group. Each group was then randomly assigned to a medicament regime. The root canal of each tooth in the experimental groups was filled with a particular preparation of intracanal medicaments as follows: Group 1—no medicament; Group 2—Calmix; Group 3—Calmix and Ledermix paste in a 50:50 mixture; Group 4—Calasept Plus and Ledermix paste in a 50:50 mixture; and Group 5—Pulpdent with the smear layer left intact.

The medicaments were placed in the root canals using a spiral filler. The order of placement in the 50:50 mix groups was Ledermix paste first and then the Ca(OH)_2_ paste; thus, the two pastes were mixed within the canal with the spiral filler. The access cavities were filled with Cavit (ESPE Dental-Medizin GmbH & Co., Seefeld, Germany), ensuring that the depth of the Cavit was at least 3 mm. The teeth were then rinsed in distilled water, returned to their storage vials, and stored at 37 °C in an incubator between all phases of the experiment.

The pH of the dentine was determined at the base of the two external cavities that had been cut into the inner and outer dentine at the following time intervals: 1, 2, 5, 7, 14, 21, and 28 days and then 5, 6, 8, and 12 weeks following placement of the medicaments. At each time interval, the teeth were removed from the storage vials and rinsed in distilled water to remove any saline residue. The cavities and the root surfaces were blotted dry, and 2 μL of distilled water was placed at the site being investigated—that is, at the base of the cavity. After 5 min, the pH of the distilled water in each cavity was measured using a pH meter with a microelectrode (Microelectrodes Inc., Bedford, NH, USA, MI—415, Batch number 77,539 and 77,540).

Two statistical models were used with the first model including time as a factor. Linear mixed models were used to establish whether or not there were any effects of the fixed factors on the pH level—that is, the medicament regime, the cavity location, and the time interval following placement. The second model used the area under the curve as the response—a linear mixed model approach was used to assess the effects on the response area under the curve with the fixed factors being the medicament regime, the cavity location, and the time interval following placement. The significance level for all statistical tests was set at the 95% confidence interval.

## 5. Conclusions

The 50:50 mix of different formulations of Ca(OH)_2_ with Ledermix paste exhibited similar release and diffusion patterns of hydroxyl ions as demonstrated by the pH measurements in the inner and outer dentine. The pH and total hydroxyl ion diffusion from these mixtures was lower than when calcium hydroxide was used alone. The diffusion patterns for Calmix alone were similar to those of other Ca(OH)_2_ pastes reported in earlier studies, but the pH levels reached in the inner and outer dentine were slightly higher. The smear layer inhibited the diffusion of hydroxyl ions, so this layer should be removed during root canal treatment in order to allow maximum medicament diffusion through the dentinal tubules for more predictable therapeutic effects.

## Figures and Tables

**Table 1:** Maximum pH values obtained and the time taken to reach maximum pH (T_max_) in the different levels of dentine.

Material	Maximum pH—Inner Dentine	Maximum pH—Outer Dentine	T_max_ pH—Inner Dentine	T_max_ pH—Outer Dentine
Control	7.45	7.46	2 days	10 weeks
Calmix	11.27	8.92	1 week	8 weeks
Calmix/Ledermix	9.42	8.98	4 weeks	8 weeks
Calasept Plus/Ledermix	9.39	8.58	4 weeks	8 weeks
Pulpdent/smear layer	9.96	8.62	5 days	8 weeks

**Table 2:** Area under the curve responses and statistical comparisons between groups.

Medicaments	Cavity Location	Estimate	Standard Error	DF	t Value	Pr > |t|
Control	Inner	602.72	4.1284	40	145.99	<0.0001
Control	Outer	606.24	4.1284	40	146.85	<0.0001
Calmix	Inner	842.84	2.9192	40	288.72	<0.0001
Calmix	Outer	694.44	2.9192	40	237.88	<0.0001
Calmix/Ledermix	Inner	748.65	2.9192	40	256.45	<0.0001
Calmix/Ledermix	Outer	676.98	2.9192	40	231.90	<0.0001
Calasept Plus/Ledermix	Inner	724.92	2.9192	40	248.33	<0.0001
Calasept Plus/Ledermix	Outer	677.28	2.9192	40	232.00	<0.0001
Pulpdent/smear layer	Inner	754.52	2.9192	40	258.47	<0.0001
Pulpdent/smear layer	Outer	664.51	2.9192	40	227.63	<0.0001
